# Recent advances in understanding and managing secondary hyperparathyroidism in chronic kidney disease

**DOI:** 10.12688/f1000research.22636.1

**Published:** 2020-09-01

**Authors:** María E. Rodríguez-Ortiz, Mariano Rodríguez

**Affiliations:** 1 Maimónides Institute for Biomedical Research (IMIBIC), Avda. Menéndez Pidal, S/N. 14004 Córdoba, Spain; 2 University of Córdoba, Avda. Medina Azahara, 5. 14071 Córdoba, Spain; 3 University Hospital Reina Sofía, Avda. Menéndez Pidal, S/N. 14004 Córdoba, Spain; 4 Spanish Renal Research Network (REDinREN), Carlos III Health Institute, Madrid, Spain; 5 Nephrology Clinical Management Unit, University Hospital Reina Sofía, Avda. Menéndez Pidal, S/N. 14004 Córdoba, Spain

**Keywords:** Chronic kidney disease-mineral and bone disorder, secondary hyperparathyroidism, calcimimetics, etelcalcetide, parathyroidectomy, chronic kidney disease, parathyroid, caSR, calcium

## Abstract

Secondary hyperparathyroidism is a complex pathology that develops as chronic kidney disease progresses. The retention of phosphorus and the reductions in calcium and vitamin D levels stimulate the synthesis and secretion of parathyroid hormone as well as the proliferation rate of parathyroid cells. Parathyroid growth is initially diffuse but it becomes nodular as the disease progresses, making the gland less susceptible to be inhibited. Although the mechanisms underlying the pathophysiology of secondary hyperparathyroidism are well known, new evidence has shed light on unknown aspects of the deregulation of parathyroid function. Secondary hyperparathyroidism is an important feature of chronic kidney disease–mineral and bone disorder and plays an important role in the development of bone disease and vascular calcification. Thus, part of the management of chronic kidney disease relies on maintaining acceptable levels of mineral metabolism parameters in an attempt to slow down or prevent the development of secondary hyperparathyroidism. Here, we will also review the latest evidence regarding several aspects of the clinical and surgical management of secondary hyperparathyroidism.

## Introduction

Mineral homeostasis is a tightly regulated process in which kidneys play an essential role. The development and progression of chronic kidney disease (CKD) cause serious disturbances in the maintenance of calcium (Ca) and phosphorus (P) metabolism. The term chronic kidney disease–mineral and bone disorder (CKD-MBD) summarizes all the alterations derived from the onset of CKD (that is, biochemical alterations, renal osteodystrophy, and extraosseous calcifications).

Parathyroid glands have a central role in mineral homeostasis. Under normal circumstances, small variations in serum Ca levels are detected by the Ca-sensing receptor (CaSR), located in the surface of parathyroid cells, which respond by secreting appropriate levels of parathyroid hormone (PTH). PTH stimulates bone resorption, releasing Ca and P ions into the extracellular space. At the renal level, PTH stimulates renal calcitriol (1,25D _3_) synthesis, which in turn promotes Ca and P absorption by the intestine. PTH, by stimulating tubular reabsorption, reduces renal excretion of Ca while exerting a phosphaturic action. All together, these actions are mainly intended to maintain appropriate concentrations of Ca. The concentration of P is regulated predominantly by fibroblast growth factor 23 (FGF23), which reduces tubular reabsorption of P. The parathyroid function is inhibited by high levels of Ca, 1,25D _3_, and FGF23. Derangements in the levels of these elements constitute potent stimuli for the onset and progression of secondary hyperparathyroidism (SHPT) ^1, 2^. Here, we will briefly review the latest advances regarding the pathophysiology and certain aspects of the management of SHPT.

## Novel insights into the pathogenesis of secondary hyperparathyroidism in patients with chronic kidney disease

The development of SHPT occurs in response to the impairment of renal function, being frequently diagnosed at advanced stages of CKD. SHPT is characterized by elevated rates of PTH synthesis and secretion accompanied by parathyroid cell hyperplasia. Classically, three main factors present in CKD have been considered responsible for the onset and progression of SHPT: hypocalcemia, hyperphosphatemia, and low 1,25D _3_ levels. Nowadays, FGF23 is also recognized as an element that is intimately involved in the pathophysiology of SHPT ^3^.

The aforementioned factors are responsible for the progression of hyperparathyroidism from diffuse to nodular by different mechanisms: hypocalcemia and hyperphosphatemia stimulates PTH production and secretion ^4– 6^ and promotes cell proliferation. Hypocalcemia increases the expression of proliferating cell nuclear antigen ^7– 9^. The absent or reduced antiproliferative action of 1,25D _3_^9^ and the impaired regulation of p21 and αKlotho/FGFR signaling by FGF23 ^10, 11^ also contribute to the development of parathyroid hyperplasia. The progression from diffuse to nodular hyperplasia is characterized by a progressive reduction in the expression of parathyroid receptors. This is known as tertiary hyperparathyroidism and at this point the gland is refractory to the action of its natural inhibitors.

In early CKD, several factors contribute to the onset of SHPT. Since early CKD, there is an increase in the renal load of P. The increase in serum PTH and FGF23 prevents the accumulation of P, but the excess of FGF23 reduces the serum concentration of 1,25D _3_, which results in a stimulus for PTH production. This effect is direct at the parathyroid level and indirect through the reduction in extracellular Ca. The increase in the tubular load of P and the progressive reduction in the glomerular filtration rate cause a reduction in the renal expression of Klotho, which generates resistance to the action of FGF23 ^12^. Thus, FGF23 production has to increase, 1,25D _3_ levels decrease further, and hyperparathyroidism is then established. With CKD stage 4, the number of nephrons is reduced to the point that the increases in PTH and FGF23 are not able to augment P excretion and hyperphosphatemia, together with hypocalcemia, develops. In animals with normal renal function, the elevation in FGF23 causes a reduction in PTH production, but as parathyroid hyperplasia develops, there is a lower expression of parathyroid FGFR and Klotho receptors, which prevent any inhibitory effect of FGF23 ^10^.

In patients on dialysis, hyperphosphatemia directly stimulates PTH secretion and maintains a persistent stimulus for parathyroid hyperplasia. Recent studies have demonstrated that high P prevents the activation of the CaSR ^13^, a mechanism whereby P stimulates PTH secretion despite normal serum Ca levels. Another important effect of P is the generation of skeletal resistance to the calcemic effect of PTH ^14^, which contributes to hypocalcemia with the subsequent stimulus for parathyroid hyperplasia. In the absence of kidney function, the production of 1,25D _3_ is very low and the consequences are hypocalcemia and parathyroid hyperplasia.

Although the mechanisms underlying the pathophysiology of SHPT are largely known, recent work has shed light on our knowledge of this pathology. Klotho serves as a co-receptor for FGF23 ^15^. However, the use of genetic approaches has revealed that there is an interaction between Klotho and the CaSR and that Klotho has a key role in suppressing PTH synthesis and parathyroid proliferation ^16^.

Several studies have shown that FGF23 suppresses PTH production by acting on the specific parathyroid receptor FGFR1 (FGF23 receptor 1)-Klotho. However, with the development of parathyroid hyperplasia, Klotho expression is reduced and the high FGF23 is not able to inhibit PTH production and parathyroid cell proliferation ^10, 11^. Clinical studies have demonstrated that FGF23 starts to rise at very early stages of CKD in an attempt to prevent accumulation of P. The increase in FGF23 is observed before the elevation in PTH ^17^. High FGF23 levels reduce the renal production of 1,25D _3_, which in turn promotes parathyroid hyperplasia. Phosphorus load, the main stimulus for FGF23 production, causes renal damage and a more rapid deterioration of renal function ^18^ and P restriction prevents the elevation in FGF23 ^19– 21^. Thus, the early control of the factors influencing FGF23 also slows down the development of SHPT.

Although evidence of a direct stimulatory effect of P on PTH secretion was demonstrated more than 20 years ago, recent work by Centeno *et al*. ^13^ shows that high P concentrations act on specific elements of the CaSR to stimulate PTH secretion. Therefore, it is very difficult to manage SHPT without controlling serum P levels. Furthermore, high serum P inhibits the calcemic response to PTH. Thus, in the presence of hyperphosphatemia, more PTH is required to maintain serum Ca levels ^14, 22^.

In regard to the signaling mechanisms involved in the development of parathyroid hyperplasia, a recent study by Kan *et al*. reported that vitamin D deficiency promotes activation of nuclear factor kappa B (NFκB) both in parathyroid glands from patients on hemodialysis and in experimental models of uremia ^23^. Given that vitamin D also inhibits NFκB in other tissues ^24^, this might represent an additional benefit of vitamin D supplementation in the management of SHPT.

It is widely known that hyperplastic glands are characterized by reduced expression of parathyroid vitamin D receptor (VDR), CaSR, and FGFR1 and its co-receptor Klotho. In this regard, a very interesting study has investigated the transcriptomic signature in hyperplastic parathyroid glands, performing comparative analyses with parathyroid adenomas and healthy tissue ^25^. In addition to the repeatedly reported down-regulation of the *VDR* and *CaSR* genes, marked reductions in *NFIL3* and *RET* were found when hyperplastic and normal tissues were compared ^25^. In addition, there is increasing awareness about the involvement of microRNAs (miRNAs) in the pathophysiology of parathyroid hyperplasia. miRNAs are small non-coding RNA molecules that modulate the expression of RNA. The miRNA profile in healthy and pathological parathyroid tissue is not well known. This matter was deeply investigated by Shilo *et al*., who found high expression of members of let-7, miR-30, and miR-141/200 miRNA families in parathyroid samples from humans, mice, and rats ^26^. In experimental hyperparathyroidism, the authors reported up-regulation of miR-29, miR-21, miR-148, miR-30, and miR-141 and down-regulation of miR-10, miR-125, and miR-25 ^26^. Taken together, these results may help unravel novel mechanisms underlying the development of SHPT as well as design strategies to prevent parathyroid hyperplasia and manage PTH secretion.

## Recent advances in the management of secondary hyperparathyroidism

### Novel calcimimetic agents

Hyperparathyroidism is associated with poor outcomes in CKD ^27^. According to the latest update of the Kidney Disease: Improving Global Outcomes (KDIGO) Guidelines on CKD-MBD, the optimal level of PTH for patients with CKD G3a-G5 not on dialysis is not known, and patients with intact PTH levels above the upper normal limit for the assay should be evaluated for factors such as hyperphosphatemia, hypocalcemia, high P intake, and vitamin D deficiency. In patients with CKD G5D requiring PTH-lowering therapy, calcimimetics, calcitriol, vitamin D analogs, or a combination of calcimimetics with calcitriol or vitamin D analogs is suggested ^28^.

The identification and cloning of the CaSR ^29^ allowed the development of calcimimetics, molecules that mimic Ca and therefore are able to modulate PTH secretion. Cinacalcet, effective in reducing PTH levels in patients on dialysis, was the first calcimimetic to be authorized for clinical use in patients on dialysis ^30, 31^. In preclinical experimental models, calcimimetics have also proven effective in preventing the development of vascular calcifications and even accelerating the regression of established calcifications in preclinical experimental models ^32, 33^. In humans, results from the ADVANCE study suggested that cinacalcet may attenuate vascular calcification in hemodialysis patients with moderate to severe SHPT ^34^. The EVOLVE trial was carried out to test the effectiveness of cinacalcet alone in reducing the risk of death or major cardiovascular events in patients undergoing dialysis, but no conclusive results were obtained ^35^. Secondary analyses of the EVOLVE study disclosed that cinacalcet significantly reduced FGF23 levels and that this reduction was associated with lower rates of cardiovascular death and major cardiovascular events ^36^. It has been described that cinacalcet increases bone mineral density when administered to patients on hemodialysis ^37^. A very recent work revealed that this effect occurs independently of changes in PTH levels ^38^.

Etelcalcetide (AMG416), a D-amino acid peptide, is a new calcimimetic recently approved in Europe and the United States for the treatment of SHPT in patients on hemodialysis. Unlike Ca and cinacalcet, etelcalcetide activates the CaSR by binding the extracellular domain ^39^. Etelcalcetide ensures better compliance ^40^ and lower pill burden than cinacalcet since it is administered at the end of the hemodialysis session. The initial recommended dose is 5 mg etelcalcetide three times a week, and the individual titration is according to the routine measurement of serum PTH and Ca levels and this represents another advantage of the use of this novel molecule.

In a phase I study, single doses of 0.5, 2, 5, and 10 mg etelcalcetide were administered to healthy volunteers, inducing rapid, dose-dependent reductions in iPTH (at 30 min), ionized Ca (at 12 h), and iFGF23 (at 24 h) and there were no gastrointestinal adverse reactions ^41^. A subsequent study performed in patients on hemodialysis reported similar results ^42^.

A randomized, double-blind multicenter clinical trial recently compared the effectiveness of intravenous (IV) etelcalcetide versus oral placebo and oral cinacalcet versus IV placebo for 26 weeks in 683 hemodialysis patients with PTH levels above 500 pg/mL ^43^. The initial doses of etelcalcetide and cinacalcet were 5 mg three times a week and 30 mg per day, respectively. The proportions of patients achieving more than 50% reduction in serum PTH were 52.4% and 40.2% in the etelcalcetide and cinacalcet arms, respectively, disclosing that etelcalcetide was non-inferior to cinacalcet in reducing PTH at the time point evaluated ^43^. The effectiveness of etelcalcetide in lowering PTH has been confirmed irrespectively of the severity of SHPT, and there were higher requirements of calcimimetic in the most severe cases of hyperparathyroidism ^44^. Overall, the effectiveness of etelcalcetide in controlling SHPT has been repeatedly proven, and a number of studies yielded similar results ^45, 46^. Furthermore, results obtained from a *post hoc* analysis of an open-label study performed in patients undergoing hemodialysis revealed that 52 weeks of etelcalcetide therapy were associated with lower circulating FGF23 and improvements in the levels of bone turnover markers ^47^.

According to a very recent work integrating data from two trials, the most frequently reported adverse events were those derived from changes in parameters of mineral metabolism (hypocalcemia, hypophosphatemia, and muscle spasm) ^48^. Safety and efficacy were evaluated following 1 year of treatment; there were no additional undesirable effects, and suppression of serum PTH levels continued ^49^.

Evocalcet is a calcimimetic agent that was developed and approved in Japan for the treatment of SHPT in patients on dialysis. It was developed in pursuit of a molecule capable of effectively managing PTH levels while avoiding the undesirable gastrointestinal effects associated with the use of cinacalcet ^50^. Several studies point out that, similarly to cinacalcet, evocalcet binds to the transmembrane domain of the CaSR ^51^.

In a phase I study, the administration of single doses of 1 to 20 mg of evocalcet produced dose-dependent changes in PTH, Ca, and P, and evocalcet had a better safety profile than cinacalcet ^52^. The effectiveness of evocalcet was confirmed in a population of 152 hemodialysis patients with SHPT. In that study, an initial dose of 1 mg of evocalcet was considered appropriate for the management of SHPT ^53^. Evocalcet has also been tested in peritoneal dialysis patients, and the results were similar in terms of efficacy and safety ^54^. Additional studies have examined the safety profile of evocalcet in patients with SHPT. The administration of either single ^55^ or multiple ^56^ doses of evocalcet effectively controlled PTH levels and was well tolerated by the patients. The long-term efficacy and safety of evocalcet have also been proven, and sustained control of PTH and low rate of gastrointestinal side effects were reported ^57^.

More recently, additional benefits of evocalcet beyond the management of phosphocalcium metabolism have been evaluated. Evocalcet has been shown to prevent extraosseous mineral deposition in an adenine-induced rat CKD model. Furthermore, evocalcet-treated animals exhibited lower rates of parathyroid cell proliferation when compared with controls ^58^.

In sum, these novel molecules constitute promising therapeutic tools for the clinical management of SHPT in terms of controlling the levels of PTH, Ca, P, or FGF23 as well as in safety and tolerability. However, additional clinical studies are needed to clarify the impact of these emerging molecules on the cardiovascular morbidity and mortality risks associated with the derangements in the levels of these elements.

## Parathyroidectomy

Parathyroidectomy (PTx) is the surgical resection of the parathyroid glands and is considered in patients with hyperparathyroidism refractory to conservative medical therapy. Owing to the availability of new molecules for medical therapy, the indications for PTx could be reduced to the following: (1) hyperparathyroidism resistant to calcimimetic administration, (2) severe refractory hyperphosphatemia, (3) severe hyperparathyroidism in dialysis without response to medical treatment, (4) cases of calciphylaxis with PTH levels above 500 pg/mL that do not rapidly respond to calcimimetics, (5) complications derived from SHPT, and (6) primary hyperparathyroidism in patients with CKD ^59^. There are different modalities of PTx: subtotal PTx, total PTx with autotransplantation, and total PTx without autografting. The choice of one or another technique will depend on the clinical characteristics of the patient and the availability of a surgeon with the skills needed to perform the procedure ^59^.

The use of PTx in patients with CKD is associated with improvements in biochemical parameters of mineral and bone metabolism ^60– 62^, cardiovascular health ^63^, and mortality ^64, 65^. However, this technique is not free of complications, such as persistent hyperparathyroidism, often due to the existence of supernumerary or abnormally located parathyroid glands ^66^, or the development of hungry bone syndrome due to PTH oversuppression ^67^.

The surgical removal of parathyroid glands is a useful resource mainly in cases of hyperparathyroidism refractory to medical treatment. However, it should be taken into account that, despite the post-surgical benefits, PTx is not free of complications, and the risks and benefits should be considered in each particular case.

## Conclusions

The management of SHPT is essential to control CKD-MBD. Thus, the appropriate prevention and treatment of SHPT may derive in lower occurrence of metabolic bone disease, vascular calcifications, and mortality. The management of SHPT is based on avoiding hyperphosphatemia and decreasing PTH levels by administrating calcimimetics and, when needed, VDR activators. PTx is required in only a small proportion of patients.
